# Nutrient density and cost of commonly consumed foods: a South African perspective

**DOI:** 10.1017/jns.2022.119

**Published:** 2023-01-25

**Authors:** Samukelisiwe S. Madlala, Jillian Hill, Ernesta Kunneke, Mieke Faber

**Affiliations:** 1 Non-Communicable Diseases Research Unit, South African Medical Research Council, Cape Town, South Africa; 2 School of Public Health, Faculty of Community and Health Sciences, University of the Western Cape, Cape Town, South Africa; 3 Department of Dietetics and Nutrition, University of the Western Cape, Cape Town, South Africa

**Keywords:** Affordability, Food price, NutrientRich Food Index, NRF9.3, South Africa

## Abstract

Food-based dietary guidelines promote consumption of a variety of nutritious foods for optimal health and prevention of chronic disease. However, adherence to these guidelines is challenging because of high food costs. The present study aimed to determine the nutrient density of foods relative to cost in South Africa, with the aim to identify foods within food groups with the best nutritional value per cost. A checklist of 116 food items was developed to record the type, unit, brand and cost of foods. Food prices were obtained from the websites of three national supermarkets and the average cost per 100 g edible portion was used to calculate cost per 100 kcal (418 kJ) for each food item. Nutrient content of the food items was obtained from the South African Food Composition Tables. Nutrient density was calculated using the Nutrient Rich Food (NRF9.3) Index. Nutrient density relative to cost was calculated as NRF9.3/price per 100 kcal. Vegetables and fruits had the highest NRF9.3 score and cost per 100 kcal. Overall, pulses had the highest nutritional value per cost. Fortified maizemeal porridge and bread had the best nutritional value per cost within the starchy food group. Foods with the least nutritional value per cost were fats, oils, foods high in fat and sugar, and foods and drinks high in sugar. Analysis of nutrient density and cost of foods can be used to develop tools to guide low-income consumers to make healthier food choices by identifying foods with the best nutritional value per cost.

## Introduction

Unhealthy diets, food choices and behaviours shaped by food environments and food systems are key contributing factors to the rise in overweight and obesity and non-communicable diseases (NCDs), which are a major public health problem worldwide^(1)^. The World Health Organisation (WHO) estimated that in 2016 more than 1⋅9 billion (39 %) adults aged 18 years and older were overweight and ≥650 million (13 %) were obese^(2)^. Overweight and obesity are important risk factors for NCDs and are caused by physical inactivity combined with excessive consumption of energy-dense foods high in fat and sugars^(2)^. High intakes of unhealthy foods such as refined grains, processed meats, ultra-processed crisps, sugar-sweetened beverages (SSB), foods high in saturated and trans fats, sweets and desserts are related to several diet-related NCDs including diabetes, cardiovascular disease, obesity and dental caries^(3,4,5)^. Globally, dietary risk is among the leading risk factors for mortality among adults^(6)^, and consuming a healthy diet is crucial for the reduction of overweight and obesity and diet-related NCDs^(7)^. In many low- and middle-income countries, diets are known to lack micronutrients especially among vulnerable groups, this could lead to the development of deficiencies in iron, zinc, folate, vitamin A, calcium and vitamin B12^(8,9)^.

South Africa, an upper middle-income country, is characterised by high rates of overweight and obesity^(10)^, with an unemployment rate of 34⋅5 %^(11)^ and 49⋅2 % of the adult population living below the upper-bound poverty line^(12)^. Diets in South Africa lack diversity^(13)^ and consumption of fruits and vegetables is low^(14)^. The South African food-based dietary guidelines (SA-FBDGs) encourage the consumption of diverse healthy food groups and emphasise the limiting of fats, sugar and salt in the diet^(15)^. However, these guidelines are difficult to follow for many South Africans due to several reasons including high food prices and inflation^(16)^. A recent report stated that COVID-19, economic decline and unemployment, and high food prices are among the key drivers for food insecurity in South Africa^(17)^. From March 2021 and March 2022, the cost of the average household food basket purchased by low-income women increased by 10⋅3 %^(18)^. The core food basket consists mainly of starches (maize meal, rice, cake flour, bread), white sugar, vegetable oil, sugar beans and chicken, tea and condiments and is not nutritionally balanced^(18)^.

The cost of food has been cited as a major determinant of dietary quality and food choices globally^(19,20)^. Healthier foods and diets are reported to be more expensive, making it difficult for people with low-income to eat a healthy nutritionally balanced diet^(19,20,21)^. In Sub-Saharan Africa, nutritious diets are the least affordable and more costly compared with other regions around the world^(21)^. Low-income groups often rely on cheaper energy-dense foods high in saturated fats, trans fats and added sugar^(21)^, which put them at greater risk of becoming overweight/obese and developing diet-related NCDs, and therefore food prices are a major contributor to inadequate diets and malnutrition^(22)^.

Identifying nutrient-dense foods with the best nutritional value per cost can potentially assist consumers to make healthier food choices. Nutrient profiling models, e.g. Nutrient Rich Food Index (NRF9.3), can be used to classify or rank foods according to their nutritional value and to identify healthier foods^(23)^. The NRF9.3 is also a useful tool to determine the relationship between the nutrient density of foods and their cost^(19,24)^, and thereby identify affordable nutrient rich foods^(25,26)^.

Approximately, 50⋅9 % deaths in South Africa are attributable to NCDs^(27)^ with diabetes accounting for 7 % of NCD-related mortality^(28)^. Due to this high prevalence, interventions such as the South African Diabetes Prevention Programme (SA-DPP) that aims to prevent the progression of diabetes and pre-diabetes in resource-poor communities in the Cape Town metropolis^(29)^ are being implemented. As part of the SA-DPP, a curriculum to promote healthier eating habits has been developed based on the SA-FBDGs. Cost of food may however hinder dietary change^(16)^ and educational tools to guide communities to make healthier food choices within their financial constraints are needed. Within this context, the present study aimed to determine the nutrient density of foods relative to cost in South Africa, with the aim to identify foods within food groups with the best nutritional value per cost.

## Methods

### Food checklist and nutrient composition

A food checklist was created based on the SA-FBDGs. Foods were grouped into the following seven major food groups: (1) starchy foods, (2) pulses (beans, peas, lentils and soya), (3) dairy, (4) fish, chicken, meat and eggs, (5) vegetables and fruits, (6) fats, oils and foods high in fat and (7) sugar and foods and drinks high in sugar. Dietary data from a validation study of the SA-DPP study (unpublished data) was used to finalise the checklist; therefore, the list contained commonly consumed foods in resource-poor communities in Cape Town, South Africa. The checklist contained raw food, prepared food and fortified products. The food type, brand name, unit and weight, and unit price per rands (ZAR) for each item was recorded on the checklist. The common or medium package size was recorded. For vegetables and fruits, weight per kg was recorded. The South African Food Composition Tables^(30)^ were used to obtain energy and nutrient content values per 100 g edible portion. For nutrient values not available in the South African Food Composition Tables, nutrient values were obtained from food manufacturing websites. Foods not considered were non-dairy creamer, diet beverages, tea, coffee, water, energy drinks, as these are mostly low calorie with little nutritional value^(26)^. The final analysis was based on a total of 116 foods representing the healthy and unhealthy groups based on the SA-FBDGs.

### Food price

Studies show that 90 % of people in Cape Town purchase food from supermarkets^(31,32)^. Therefore, retail food prices for the food items were obtained online from the national websites of three national supermarkets namely, Pick n Pay, Checkers and Shoprite. In-store visits were done for products that were not available online. Prices were collected between September 2020 and February 2021 to account for seasonal availability of certain fruits and vegetables. Food prices were collected for Shoprite first, which generally is cheaper than the other two supermarkets. For packaged food, the price for the brand with the lowest cost was collected. For the other two supermarkets, the price for the same brand used for Shoprite was collected. Only regular prices were recorded, not sale/promotional pricing. Food prices were recorded in ZAR ($0⋅06). For each food item, the average of the prices collected from the supermarkets was used to calculate the cost (ZAR) per 100 g edible portion using yield factor and retention factors to adjust for preparation and waste^(33)^, which was then used to calculate cost per 100 kcal. Energy density was calculated per 100 g edible portion and per 100 kcal.

### Nutrient density

Nutrient density is defined as the ratio of nutrient content to total energy. Calculations based on 100 kcal rather than 100 g, nutrient density is better reflected^(24)^. The nutrient density for each of the food items was calculated using the Nutrient Rich Foods Index NRF9.3 model^(34)^. The NRF9.3 was based on the subtraction of two subscores: Nutrients to encourage (NRn) subscore minus nutrients to limit (LIM) subscore. The NRn subscore is the sum of the percentages of daily values (DVs) of protein, fibre, vitamin A, vitamin B6, vitamin D, folate, calcium, zinc and iron. The LIM subscore is the sum of the percentages of the maximum recommended values (MRVs) of saturated fat, added sugar and sodium^(8,34)^. The reference DV and MRV were based on the FAO Codex nutrient reference values^(35)^ and are summarised in Table 1. Percentages of DV were capped at 100 % to avoid the index score to be disproportionately effected by one nutrient present in very large amounts^(8)^. The US Food and Drug Administration guidelines were used to determine nutrients selected for the model^(9)^. Nutrients of public health concern among South African adults were included in the model. The nutrients reported to be low in the diet of South African adults are vitamin A, vitamin D, folate, iron, zinc^(36)^, calcium and vitamin B6^(37)^. Nutrients to limit were selected following the guidance of previous studies^(8,34)^.

**Table 1.** Reference daily values and maximum recommended values for nutrients

Nutrients	Standard
Protein (g)	50
Fibre (g)	25
Vitamin A (μg RE)	800
Vitamin B6 (mg)	1⋅3
Vitamin D (μg)	5
Folate (μg)	400
Calcium (mg)	1000
Zinc (mg)	14
Iron (mg)	22
Maximum recommended values	
Saturated fat (g)	22
Added sugar (g)	50
Sodium (mg)	2000

g, grams; μg, micrograms; RE, retinol equivalents; mg, milligrams.

Reference values from the FAO Codex nutrient reference values^(35)^.

The NRF9.3 Index score was calculated per 100 kcal and per 100 g for each food item. The nutrient-to-price ratio (NPR) was used as an indicator for foods with the best nutritional value per cost and was calculated by dividing the NRF9.3 score to cost (ZAR) per 100 g and cost (ZAR) per 100 kcal of food. Foods were ranked according to the NRF9.3 score per 100 kcal, and NPR.

### Data analysis

Data were captured into Microsoft Excel data files. All analyses were performed using IBM SPSS for Windows version 28 (Armonk, New York, USA). The Shapiro–Wilk test was performed to test the data for normality. Continuous data were expressed as median and interquartile range (IQR). Median (IQR) values of the NRF9.3 (per 100 kcal and per 100 g), energy density (kcal/100 g), food prices (ZAR/100 g and ZAR/100 kcal) and NPR for each food item and food group were computed. Analysis of variance (ANOVA) test was used to compare energy density, nutrient density and NPR across food groups. The Tukey *post hoc* test was used to locate differences between food groups. Bubble/Scatter plots were used to show the relationship between nutrient density and energy density, cost per 100 kcal and NPR. Spearman correlation analysis was performed to assess the relationship between the NRF9.3 score and the cost per 100 kcal of foods. Significance was set at *P*-value < 0⋅05.

## Results

Table 2 shows the median energy density, nutrient density (based on the NRF9.3 score), cost and NPR (per 100 g and per 100 kcal) for 116 food items grouped into 7 food groups. *Post hoc* analysis showed that there were significant differences between food groups. Energy density was lowest for the vegetables and fruits group (52⋅4 kcal/100 g), and highest for fats, oils and foods high in fat group (573⋅4 kcal/100 g). Nutrient density was highest for the vegetables and fruits group, followed by pulses, and was lowest for the sugar and foods and drinks high in sugar group. Cost per 100 g was highest for the fish, chicken, meat and eggs group (ZAR 10⋅9/100 g) and lowest for the pulses and starchy foods groups (ZAR 1⋅6/100 g). Cost per 100 kcal was highest for the vegetables and fruits group (ZAR 7⋅7/100 kcal), followed by the fish, chicken, meat and eggs group (ZAR 4⋅8/100 kcal) and the dairy group (ZAR 3⋅3/100 kcal).

**Table 2.** Median energy density, nutrient density (NRF9.3), food prices (per 100 g and per 100 kcal) and nutrient-to-price ratio of food groups

	*N*	Energy density (kcal/100 g)	Nutrient density (NRF9.3/100 kcal)	Price per 100 g	Price per 100 kcal	NPR NRF9.3/ZAR 100 g	NPR NRF9.3/ZAR 100 kcal
		Median (IQR)	Median (IQR)	Median (IQR)	Median (IQR)	Median (IQR)	Median (IQR)
All items	116	142·7 (62·8–373·1)	30·9 (12·5–68·0)	3·9 (2·0–7·9)	2·6 (1·4–5·5)	8·2 (3·1–24·2)	8·3 (3·1–24·8)
Food groups							
Starchy foods^a^	20	136·5 (83·2–340·4)^e^^,^^f^	35·2 (26·6–47·9)^e^^,^^g^	1·6 (0·6–3·3)^d^^,^^f^	0·8 (0·5–1·6)^e^	41·3 (24·7–85·3)^c^^,^^d^^,^^e^^,^^f^^,^^g^	43·5 (24·7–95·4)^c^^,^^d^^,^^e^^,^^f^^,^^g^
Pulses^b^	5	120·7 (103·9–134·2)^f^	77·7 (55·7–115·3)^f^^,^^g^	1·6 (1·4–3·2)^d^	1·2 (1·1–3·1)^e^	67·1 (24·2–77·8)^f^^,^^g^	67·1 (24·2–77·8)^g^
Dairy^c^	10	76·9 (62·1–339·2)^f^	32·3 (26·3–41·9)^e^^,^^g^	3·0 (1·3–14·2)	3·3 (2·1–4·2)^e^	11·9 (6·4–16·1)^a^	11·9 (6·4–16·1)^a^^,^^g^
Fish, chicken, meat and eggs^d^	20	221·8 (150·6–304·9)^e^^,^^f^	41·8 (16·3–92·6)^f^^,^^g^	10·9 (6·1–16·9)^a^^,^^b^^,^^e^^,^^g^	4·8 (3·1–6·9)^a^^,^^b^	7·9 (2·5–9·7)^a^	7·9 (2·5–9·7)^a^
Vegetables and fruits^e^	30	52·4 (27·4–64·1)^a^^,^^f^^,^^g^	82·9 (33·3–132·6)^a^^,^^c^^,^^f^^,^^g^	3·2 (2·0–3·2)^d^	7·7 (3·5–12·8)^d^^,^^f^^,^^g^	8·7 (4·7–17·1)^a^^,^^g^	8·7 (4·7–15·4)^a^^,^^g^
Fats, oils and foods high in fat^f^	18	573·4 (410·3–714·5)^a^^,^^b^^,^^c^^,^^d^^,^^e^^,^^g^	10·1 (−4·1–15·8)^b^^,^^d^^,^^e^	7·8 (3·5–10·1)^a^	1·6 (0·6–2·2)^e^	4·5 (−2·5–8·8)^a^	5·7 (−5·2–11·2)^a^
Sugar & foods and drinks high in sugar^g^	13	364·1 (134·5–433·3)^e^^,^^f^	−34·1 (−72·1–(−11·8))^a^^,^^b^^,^^c^^,^^d^^,^^e^	4·6 (1·9–6·6)^d^	1·5 (1·4–2·9)^e^	−9·6 (−29·6–(−4·5))^a^^,^^b^^,^^e^	−9·6 (−42·4–(−6·6))^a^^,^^b^^,^^c^^,^^d^^,^^e^^,^^f^
*P*-value*		<0·001	<0·001	<0·001	<0·001	<0·001	<0·001

NRF, Nutrient Rich Foods; NPR, nutrient-to-price ratio; ZAR, South African Rand; IQR, interquartile range; 100 kcal = 418 kJ.

* Statistical difference between food groups, obtained by ANOVA test, significant at *P* < 0·001 level.

Each food group (the reference food group) was assigned a letter.

a Starchy foods.

b Pulses.

c Dairy.

d Fish, chicken, meat and eggs.

e Vegetables and fruits.

f Fats, oils and foods high in fat.

g Sugar and foods and drinks high in sugar. Median superscript letters indicate food groups that differ significantly from the reference food group; ANOVA Tukey *post hoc* test, significant at *P* < 0·05 level.

**Fig. 1.**
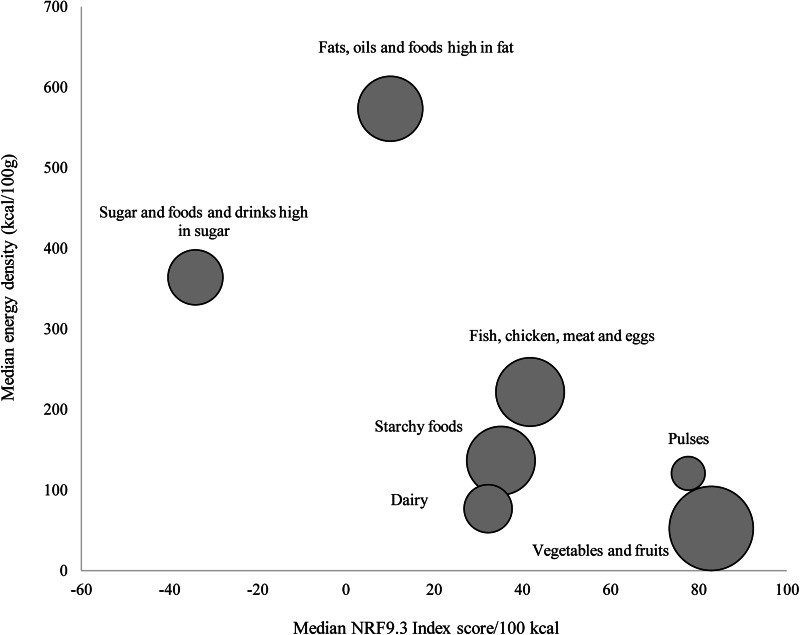
Median Nutrient Rrich Foods (NRF9.3) scores in relation to energy density (kcal/100 g) for seven major food groups.

Fig. 1 shows the relation between median nutrient density and energy density of food groups. The fats, oils and foods high in fat group had the highest energy density but a low nutrient density score. The vegetables and fruits group had the highest nutrient density score but the lowest energy density.

**Fig. 2.**
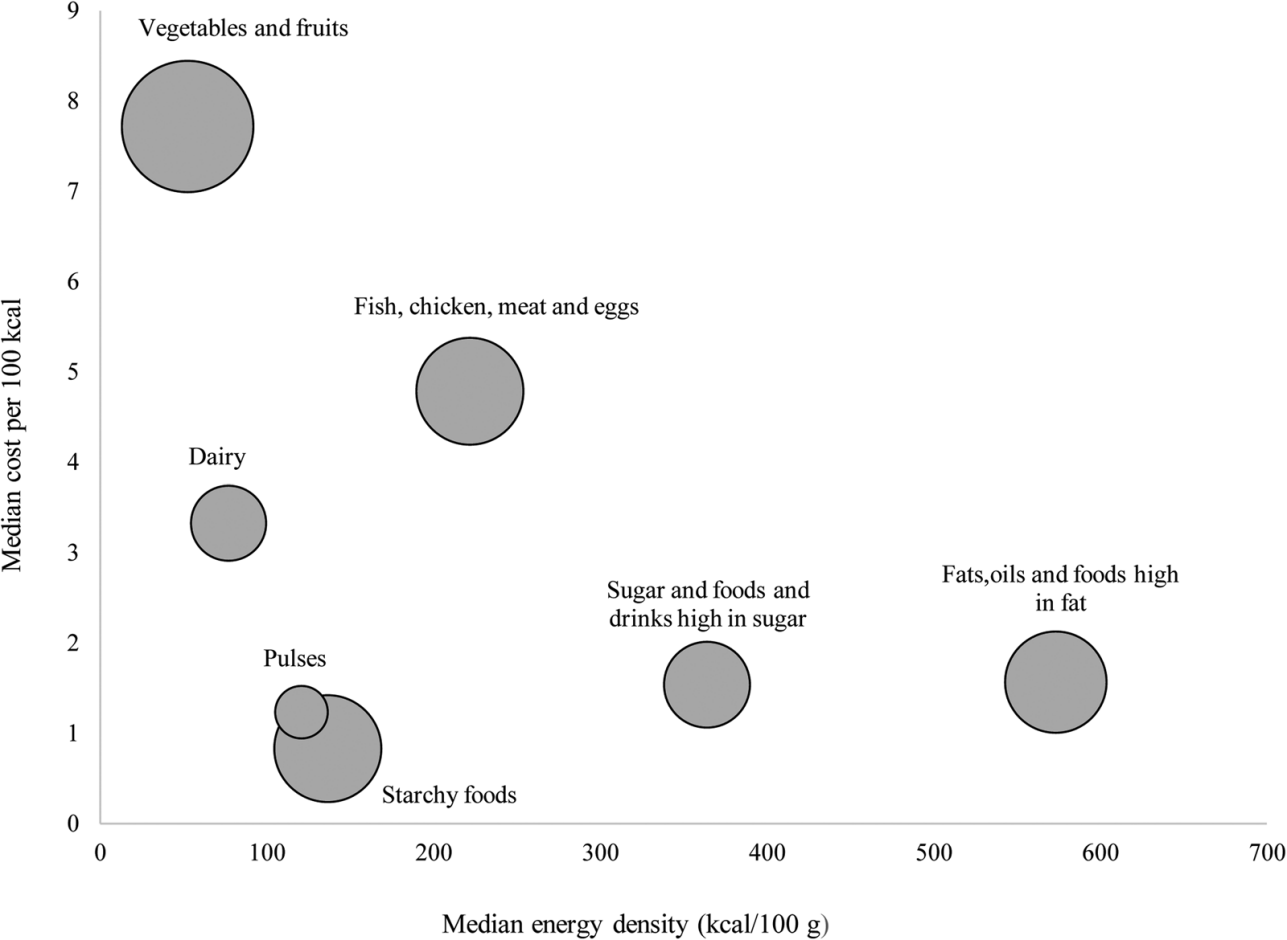
Median energy density (kcal/100 g) in relation to cost per 100 kcal by seven major food group.

Fig. 2 shows the relation between median energy density in relation to cost per 100 kcal for food groups. The fats, oils and foods high in fat group, sugar and foods and drinks high in sugar group as well as starchy foods had the lowest cost less per 100 kcal and are therefore the cheapest sources of energy. The vegetables and fruits group had a high nutrient density and cost more per 100 kcal in comparison to other food groups. The pulses group had a lower cost per 100 kcal but high nutrient density. The ranking of individual foods according to the energy-to-cost ratio is indicated in Supplementary Table S1. Healthier foods such as vegetables and fruits, lean meat, fish and chicken were the most expensive sources of energy.

**Fig. 3.**
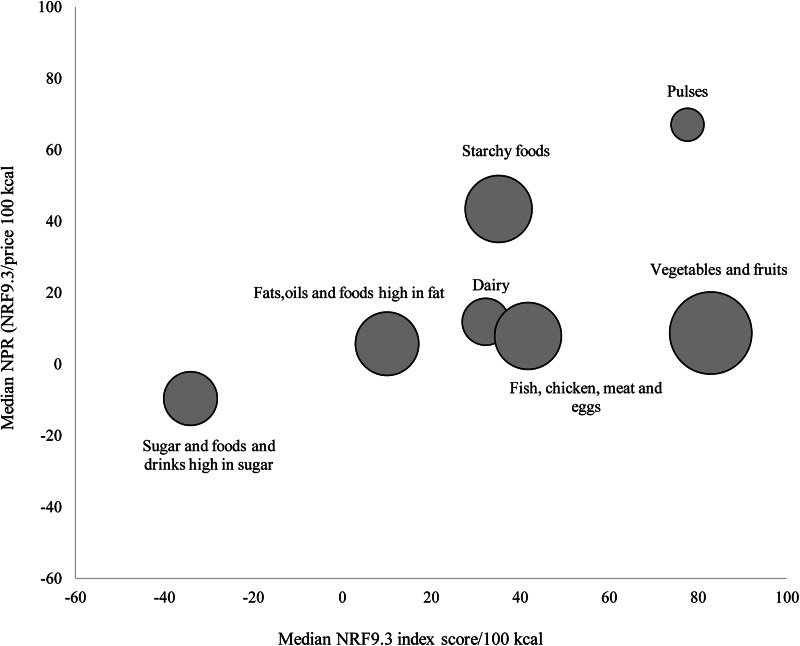
Median Nutrient Rich Foods (NRF9.3) scores shown in relation to the nutrient-to-price ratio (NPR) (NRF9.3/price 100 kcal) by seven major food groups.

Fig. 3 shows the relation between median nutrient density scores and NPR (per 100 kcal) of food groups. Food groups with the highest median NPR (per 100 kcal) were pulses and starchy foods, while the sugar and foods and drinks high in sugar group had the lowest median NPR (per 100 kcal).

**Fig. 4.**
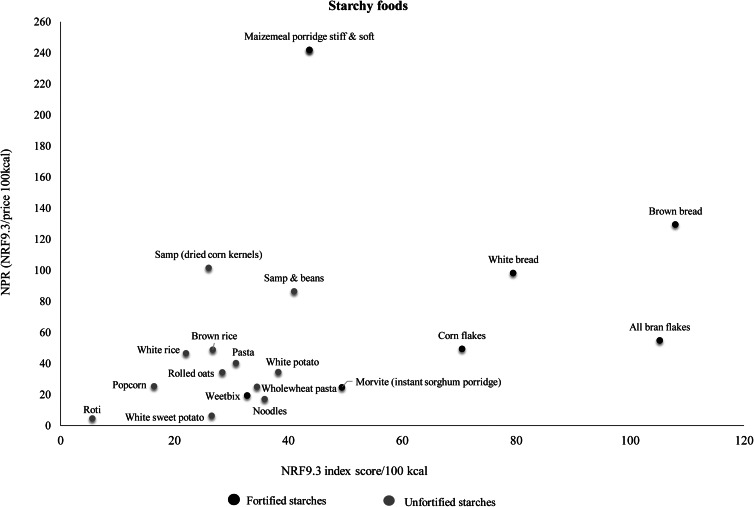
Relation of the nutrient-to-price ratio (NPR) and the nutrient density (NRF9.3) score for starchy foods.

**Fig. 5.**
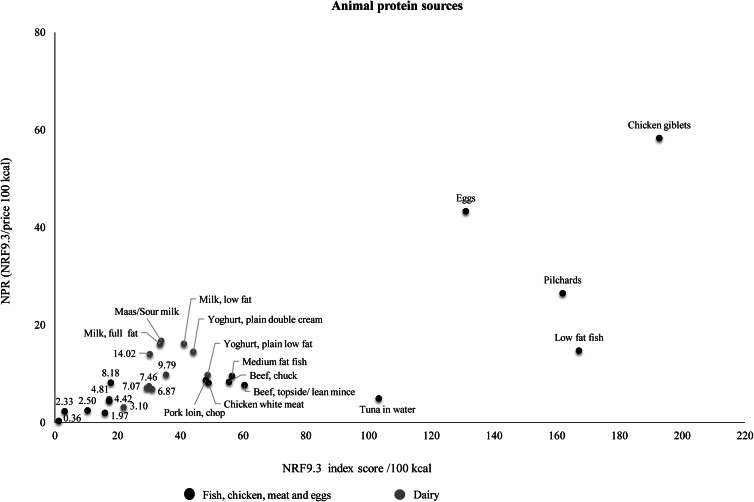
Relation of the nutrient-to-price ratio (NPR) and the nutrient density (NRF9.3) score for animal protein sources.

**Fig. 6.**
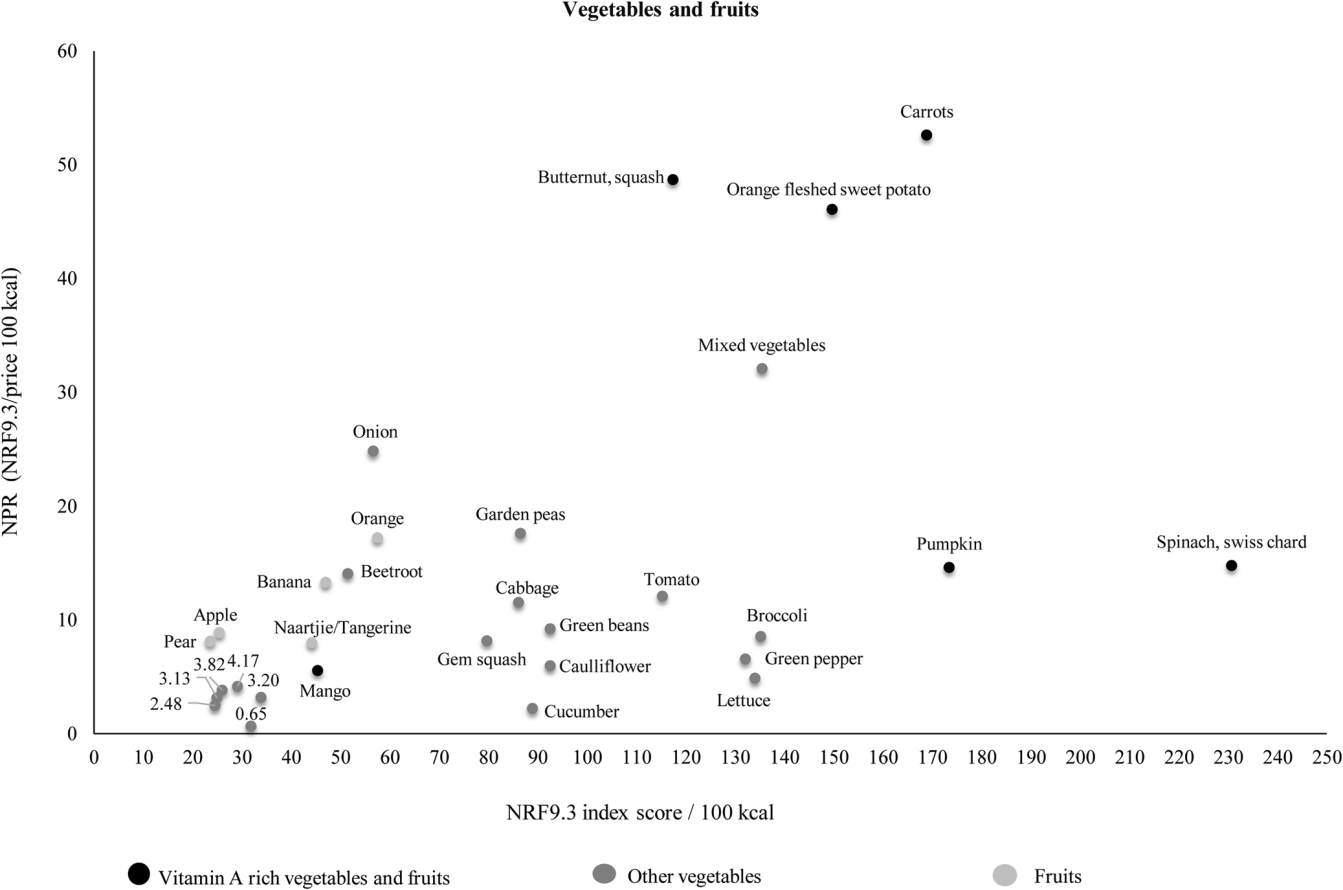
Relation of the nutrient-to-price ratio (NPR) and the nutrient density (NRF9.3) score for vegetables and fruits.

Table 3 shows the ranking of foods within food groups according to NPR (per 100 kcal). In Table 3, two subgroups are given for the starchy food group (fortified and unfortified starch foods) and three subgroups for the vegetables and fruits group (vitamin A-rich vegetables and fruits, other vegetables and other fruits). Fortified starches, particularly maize meal and to a lesser extent bread, had higher NPR values than unfortified starches. Pulses with the highest NPR values were lentils, sugar beans and split peas. Dairy products had lower NPRs compared with the fish, chicken, meat and eggs group. Chicken giblets, eggs, pilchards and low-fat fish had the best nutrient density relative to cost. Dairy products with the highest NPR values were sour milk, low fat milk, full cream milk and double cream yoghurt. Vitamin A-rich vegetables and fruits had higher NRF9.3 scores compared with other fruits and vegetables. Vegetables with the highest NPR were carrot, butternut, orange-fleshed sweet potato and mixed vegetables. Figs. 4–6 show the relation of NPR (per 100 kcal) and the nutrient density score for starchy foods, animal protein sources and vegetables and fruits, respectively.

**Table 3.** Ranking of selected South African foods within each food group according to the nutrient-to-price ratio per 100 kcal.

Food groups	Food item	NPR (NRF9.3/ZAR 100 kcal)	Nutrient density (NRF9.3/100 kcal)	Price per 100 kcal
Fortified starchy foods	Stiff porridge (maize meal, fortified)	242	44	0·18
	Soft porridge (maize meal, fortified)	242	44	0·18
	Brown bread (fortified)	129	108	0·83
	White bread (fortified)	98	79	0·81
	All bran flakes, breakfast cereal	55	105	1·92
	Corn flakes, plain, breakfast cereal	49	71	1·43
	Morvite original instant porridge, prepared^¶^	25	49	2·01
	Weet-Bix, breakfast cereal	19	33	1·70
Unfortified starchy foods	Samp, cooked (white)*	102	26	0·26
	Samp and beans, 1:1, cooked	87	41	0·47
	Brown rice, cooked	49	27	0·55
	White rice, cooked	47	22	0·47
	Pasta, Macaroni/Spaghetti, cooked	40	31	0·77
	Potato, boiled without skin	34	38	1·11
	Oats, rolled, cooked	34	28	0·83
	Popcorn, plain	25	16	0·65
	Pasta, whole wheat Macaroni/Spaghetti, cooked	25	34	1·38
	Noodles, egg, cooked	17	36	2·11
	White-fleshed sweet potato, boiled	6	27	4·15
	Roti, made with sun oil	5	6	1·21
Pulses	Lentils, whole, cooked	85	104	1·23
	Sugar beans, cooked	71	78	1·09
	Lentils, split	67	73	1·09
	Soya mince, cooked	32	126	3·95
	Baked beans, canned in tomato sauce	17	38	2·31
Dairy	Maas/Sour milk	17	34	2·02
	Milk, low fat/2 % fat, fresh	16	41	2·55
	Milk, full fat/whole, fresh	16	34	2·08
	Yoghurt, plain, double cream	15	44	3·05
	Milk, full fat/whole, UHT	14	30	2·16
	Yoghurt, plain, low fat	10	49	5·00
	Cheese, Cheddar	7	29	4·15
	Cheese, Gouda (Edam, Swiss)	7	31	4·51
	Yoghurt, fruit, low fat, sweetened	5	17	3·61
	Cheese, processed, full fat	3	10	4·17
Fish, chicken, meat and eggs	Chicken giblets, cooked	58	193	3·30
	Egg, chicken, boiled/poached	43	131	3·02
	Pilchards in tomato sauce, canned	27	162	6·10
	Fish, low fat, grilled	15	167	11·33
	Chicken, meat and skin, frozen, roasted	10	35	3·62
	Fish, medium fat, grilled/steamed	10	57	5·91
	Pork, loin, grilled (chop)	9	48	5·55
	Beef, chuck, cooked – moist	8	56	6·65
	Chicken, feet, raw	8	18	2·18
	Chicken, white meat, fresh, cooked	8	49	6·02
	Beef, topside/lean mince, cooked	8	60	7·87
	Patty, beef, frozen, grilled	7	30	4·02
	Tuna, canned in water	5	103	20·97
	Beef, brisket/regular mince, cooked	4	17	3·91
	Mutton, shoulder, braised	3	22	7·08
	Polony/Bologna, beef and pork	2	3	1·34
	Mutton, loin, grilled (chop)	2	16	8·12
	Bacon, cured, pan-fried/grilled	0·4	1	3·53
	Vienna sausage, beef and pork, canned^§^	−3	−8	2·46
	Sausage, beef and pork/boerewors, grilled	−4	−10	2·65
Vitamin A-rich fruits and vegetables	Carrot, boiled (flesh and skin)	53	169	3·21
	Butternut, squash, boiled	49	117	2·41
	Orange-fleshed sweet potato, baked	46	150	3·25
	Spinach (Swiss Chard), boiled	15	231	15·58
	Pumpkin, boiled	15	173	11·85
	Mango, raw (peeled)	6	45	8·14
	Peach, raw	4	29	6·99
Other vegetables	Mixed vegetables, frozen, boiled	32	135	4·22
	Onion, boiled	25	57	2·28
	Peas, frozen, boiled	18	86	4·91
	Beetroot, boiled with skin	14	51	3·66
	Tomato, raw	12	115	9·54
	Cabbage, boiled	12	86	7·47
	Green beans, frozen, boiled	9	92	10·03
	Broccoli, boiled	9	135	15·80
	Gem squash, boiled	8	80	9·75
	Pepper, sweet, green, boiled	7	132	20·14
	Cauliflower, boiled	6	92	15·47
	Lettuce, raw	5	134	27·52
	Cucumber, English, raw	2	89	40·16
Other fruits	Orange, raw (peeled)	17	57	3·34
	Banana, raw (peeled)	13	47	3·52
	Apple, golden delicious, raw	9	25	2·85
	Pear, raw	8	24	2·89
	Naartjie/Tangerine, raw (peeled)	8	44	5·51
	Avocado, raw (peeled)	5	13	2·58
	Plum, raw	4	26	6·82
	Mango and orange juice	3	34	10·59
	Nectarine, raw	3	25	7·96
	Grape, average, raw	2	26	9·90
	Pineapple, raw (peeled)	0·7	32	48·98
Fats and oils	Margarine, brick/hard	39	15	0·37
	Margarine, polyunsaturated, soft	38	15	0·40
	Salad dressing, French	−3	−5	1·51
	Canola oil	−11	−4	0·35
	Butter	−12	−20	1·63
	Salad dressing, mayonnaise	−16	−11	0·70
	Sunflower oil	−18	−5	0·30
Foods high in fat	Vetkoek, home-made^≠^	14	25	1·73
	Snack, savoury, potato crisps/chips	12	29	2·41
	Pie, chicken, commercial, baked	11	23	2·10
	Peanut butter (unsalted/unsweetened)	8	12	1·48
	Peanuts, roasted, salted	8	17	2·19
	Peanut butter, smooth style	7	8	1·16
	Avocado, raw (peeled)	5	13	2·58
	Potato chips/French fries	6	12	1·92
	Snack, savoury, average, e.g. Niknaks, Fritos^ǂ^	1	1	1·46
	Samoosa, with mutton filling	−1	−3	2·67
Sugar and foods and drinks high in sugar	Dairy-fruit juice mix	7	18	2·65
	Muffin, plain	5	8	1·50
	Doughnut, plain	−1	−2	2·25
	Cold drink, squash, diluted	−6	−45	7·58
	Sweets, fruit gum	−7	−34	4·79
	Cookies, commercial, plain	−8	−11	1·40
	Sweets, chocolate, milk	−8	−25	3·05
	Ice cream, regular (10 % fat)	−10	−13	1·34
	Cookies, commercial, with filling	−13	−18	1·40
	Jam/Marmalade	−22	−40	1·81
	Sweets, hard boiled and soft jelly type	−31	−48	1·54
	Cold drink, carbonated	−54	−98	1·83
	Sugar, brown	−189	−97	0·51
	Sugar, white, granulated	−213	−100	0·47

NPR, nutrient-to-price; NRF, Nutrient Rrich Foods; ZAR, South African Rand, 100 kcal = 418 kJ.

¶ Morvite – instant sorghum porridge.

* Samp – dried corn kernels.

§ Vienna sausage – Hot dog/Frankfurter (thin parboiled sausage traditionally made of pork and beef).

≠ Vetkoek – Fried dough bread.

ǂ Niknaks, Fritos – Corn-based snack.

The ranking of individual foods by NPR (per 100 kcal) and NPR (per 100 g) are indicated in Supplementary Tables S2 and S3. The top 50 foods ranked included a mixture of food items, but it was dominated by starchy foods. Overall, energy-dense foods had higher cost per 100 g than per 100 kcal (Supplementary Table S2). Spearman correlation analysis showed that the nutrient density score is positively related to the cost per 100 kcal of food item (*r* = 0·434, *P* = <0·01), indicating that when nutrient density increases so does the energy cost of food.

## Discussion

The findings of the present study suggest that energy density, nutrient density, cost and nutrient density relative to cost varies across and within food groups. Based on the NRF9.3 scores, the vegetable and fruit group had the highest nutrient density, followed by pulses, fish, chicken, meat and eggs group, starchy foods and the dairy food group. Overall, vegetables and fruits also had the highest cost per 100 kcal in comparison with the other food groups, and are therefore the most nutrient-dense but also the most expensive per 100 kcal. Nutritional value per cost was highest in the pulses food group. Fats, oils and foods high in fat, and sugar and foods and drinks high in sugar had the highest energy density and lowest nutritional value per cost and were therefore the most affordable sources of energy however they were not nutrient rich.

The starchy food group had the second best nutritional value per cost in comparison with other food groups. This is in contrast to a Brazilian study which showed that starchy foods (grains and cereals) had the lowest nutritional value per cost^(38)^. In South Africa, mandatory fortification of two staple foods, maize meal and bread flour, was introduced in 2003 to improve nutrient intakes and address micronutrient deficiencies in the population^(39)^. These fortified staple foods, which are widely consumed in South Africa, had the best nutritional value per cost within the starchy food group^(40)^. Starchy foods overall had the lowest energy cost, which is in line with literature stating that starches and grains are the cheapest source of energy^(38,41)^. The SA-FBDGs recommend that starchy foods be included in most meals^(15)^, but excessive consumption of these high energy refined starches may lead to overweight and obesity^(42,43)^.

The fish, chicken, meat and eggs group had a relatively high nutrient density score, but had the fourth highest nutritional value per cost with dairy foods having the third highest. Chicken giblets, eggs, canned pilchards and milk (including low and full fat milk), respectively had the highest nutritional value per cost of animal protein sources. Similarly, a French study found that organ meat had the highest nutritional value per cost in the meat group, and eggs also had a high nutritional value per cost^(25)^. According to another French study, organ meats, beef, eggs, milk, canned fish with bones, lamb/mutton, and cheese had the highest micronutrient density of all animal protein sources, while deli meats had the lowest nutrient density score in the meat group^(44)^. Our results show that processed meat such as polony, viennas and sausages are cheaper animal-source foods, but their nutrient density is also very low. Processed meat in South Africa is less expensive in comparison with red meat and chicken and may be more preferred by people with lower income^(45)^. There is limited data on the consumption of processed meat, however FAOSTAT balance sheets between 1999 and 2009 show that processed meat consumption increased by 45·8 %^(46)^. Processed meat is classified as carcinogenic and consumption of processed meat is associated with colorectal cancer^(47)^. The eighth SA-FBDG states that fish, chicken, lean meat and eggs can be eaten daily. It is important for consumers to continue to be educated about the benefits of eating lean meats and be encouraged to consume these foods in moderation, particularly as the consumption of meat and processed meat increases in South Africa^(45)^.

Pulses had the best nutritional value per cost across all food groups. These foods are good source of carbohydrates, protein, fibre and several micronutrients including iron, magnesium and potassium and are therefore known to be nutrient rich^(48)^. Pulses may be beneficial in preventing and managing NCDs as they can potentially reduce the risk of obesity^(49)^ and certain cancers^(50)^. Since pulses have a much lower cost per 100 kcal in comparison with animal protein sources and have a higher nutrient density relative to cost, they would be a good protein substitute and would be a more affordable choice for low-income consumers. Although using pulses as a meat substitute is encouraged in the SA-FBDGs^(51)^, the recommended intake is lower than the Eat Lancet recommendations^(52)^. It has been suggested that promotion of legumes and soya be included in the National Food and Nutrition Security communication plan, as this may stimulate production and consumption of these foods^(52)^.

Overall, the vegetables and fruits group had the highest nutrient density but also the highest energy cost. Within the vegetables and fruits group, vitamin A-rich fruits and vegetables had the highest NRF9.3 scores, which is similar to findings of a study that was done in New Zealand^(53)^. In contrast to studies in Brazil^(38)^ and New Zealand^(53)^, which reported high nutrient-to-cost ratios, the vegetables and fruits group had a low NPR in our study. Generally, vegetables and fruits are reported to be expensive^(21)^, and cost has been cited globally as a major barrier to acquiring vegetables and fruits^(54)^. Although vegetables and fruits are VAT zero-rated in South Africa^(55)^, cost prevents consumption of these foods among low-income households^(56)^. The South African population consumes less than half of the WHO recommended daily intake of 400 g for the prevention of cardiovascular diseases and some types of cancers^(11)^. Low-income consumers are concerned about getting the most kilojoules per unit cost^(52)^, and it may therefore be difficult to advise them to eat more vegetables and fruits^(57)^. Home gardening and community gardens have been shown to improve the availability and access to a variety of vegetables and improve dietary diversity and overall dietary intake among children and adults in urban and rural communities in South Africa^(58,59)^. Households should thus be encouraged to grow at least some vegetables and/or fruits. Furthermore, vegetables and fruits had the lowest energy density and therefore supply a significant amount of nutrients for fewer calories^(60)^. Considering the high rates of overweight and obesity in South Africa^(10)^, consumption of high-water content vegetables and fruits should be encouraged to aid in reducing calorie intake and therefore curb overweight and obesity.

Numerous studies have shown that energy density and energy cost are inversely related, suggesting that higher food price is associated with lower energy density^(61,62)^. The fats, oils and foods high in fat group, and the sugar and foods and drinks high in sugar group had the highest energy density, lowest nutrient density, lowest energy cost and lowest nutrient density relative to cost. These findings are supported by literature that states that fats and sweets are the cheapest sources of energy^(24,38,44,63)^. Sugar consumption in South Africa exceeds the WHO recommendations of total energy intake (<10 %)^(64)^. In an attempt to reduce sugar and calorie intake, and purchasing of SSBs, a levy tax on SSBs was introduced in 2016 in South Africa^(65)^. However, these high sugar drinks are still relatively low cost. The SA-FBDGs emphasise that fats and oils, sugar and foods and drinks high in sugar should be used sparingly^(15,66)^, as overconsumption of nutrient poor foods is associated with weight gain and subsequent negative health outcomes such as diabetes^(52)^.

Food price and diet cost are known to limit access to healthy diets among low-income consumers^(44)^. People tend to consume foods that they can afford to purchase^(67)^, and food cost therefore contributes to lower-income groups’ inability to adhere to dietary guidelines^(68)^. Low-cost energy-dense foods are more accessible for low-income households, which contributes to overweight and obesity in low-income settings^(69)^. Also, besides from being cheaper, unhealthy foods are often convenient and highly palatable compared with healthier foods which often require preparation skills and are less palatable^(42)^. Increasing the price of nutrient poor foods through taxation has been shown to reduce the purchasing of such foods^(70)^. Other barriers to purchasing healthier foods besides cost include accessibility, food distribution and retail, food storage, food preservation and safety, cooking skills or preparation time^(57)^.

A healthy diet is often unaffordable for the majority of the South African population^(71)^. Low-income households in Limpopo province in South Africa were reported to use various strategies to combat rising food prices; these included eating indigenous or traditional foods and growing vegetables at home^(72)^. Agricultural interventions can improve not only livelihoods, but household food security as well^(73)^. These interventions could benefit mostly low-income consumers who cannot afford to buy fresh vegetables and fruits. It has been argued that changes in the food and agricultural sector are needed to improve the South African food system^(74)^, that changes to agricultural policies and store policies can improve access to quality and affordable diets^(75)^, and that the implementation of food assistance programmes may be a viable short-term strategy that can lower the cost of nutritionally balanced diets^(25)^.

A strength of the present study is that the average price was calculated for each food item to account for food price variation across three stores. However, the study was based on 116 food items and did not include all foods available in national supermarkets. All foods selected in the study are however available nationally. The food checklist was based on foods commonly consumed by low-income households in Cape Town and is therefore not representative of all foods eaten in South Africa. Also, the collected food prices were limited to three supermarkets mostly used by low-income households in the Western Cape province; these supermarkets do however represent the main food chains in South Africa. The cost of food items was not recorded for all brands available, but the lowest priced supermarket (Shoprite) was used to determine the lowest priced brands for which information was then collected in all three supermarkets. The NRF Index calculations were limited to selected nine macronutrients, vitamins and minerals, if different nutrients are used, the results may vary. Food prices collected were limited to Western Cape province. Food prices may however differ by province/geographical location and seasonality.

## Conclusion

Through nutrient profiling, the study identified foods within food groups with the best nutritional value per cost. Food groups with the best nutritional value per cost were pulses, starchy foods, dairy, vegetables and fruits, and fish, chicken, meat and eggs, respectively. Pulses such as sugar beans and lentils had the best nutritional value per cost and would be a more affordable substitute for meat and chicken for low-income consumers. The FBDGs recommend eating vegetables and fruits daily, yet these foods, although nutrient dense, were also the most expensive sources of energy. In an environment of rising food prices, South African households can increase vegetable and fruit consumption through home and community gardens. Compared with other studies done on the nutrient density of foods, our study included fortified staple foods which were found to have the highest nutritional value per cost within the starchy foods group. Fortification of staple foods can provide nutritional benefits at low costs, particularly for low-income consumers who rely on these foods during times of financial difficulties. The food groups with the least nutritional value per cost were fats, oils and foods high in fat and sugar and foods and drinks high in sugar; these foods were also the cheapest sources of energy and therefore should be consumed sparingly as stated in the FBDGs. This research can be used in public health interventions to prevent micronutrient deficiencies and reduce the burden of disease among people with lesser financial means. The identification of foods with the best nutritional value per cost can be used to develop public health educational tools to guide consumers in making healthier food choices and encouraging adherence to FBDGs in resource-poor settings.

## Supplementary material

For supplementary material accompanying this paper visit https://doi.org/10.1017/jns.2022.119. click here to view supplementary material
