# Supplementary material for: Nutrient density and cost of commonly consumed foods: a South African perspective
Source: J Nutr Sci. 2023 Jan 25;12:e10. doi: 10.1017/jns.2022.119 (PMC9879879; doi:10.1017/jns.2022.119)
Supplement: Supplementary file 1 [file S2048679022001197sup001.docx]

**Table S1**. Ranking selected South African foods according to price per 100kcal. Also shown are energy density and NRF9.3 scores

| No. | Food | Energy cost (ZAR/100kcal) | Energy density (kcal/100g) | Nutrient density (NRF9.3/100kcal) |
| --- | --- | --- | --- | --- |
| 1 | Stiff porridge (maize meal-fortified) | 0.18 | 150 | 44 |
| 2 | Soft porridge (maize meal-fortified) | 0.18 | 64 | 44 |
| 3 | Samp, cooked (white)^*^ | 0.26 | 105 | 26 |
| 4 | Sunflower oil | 0.30 | 884 | -5 |
| 5 | Canola oil | 0.35 | 884 | -4 |
| 6 | Margarine, brick/hard | 0.37 | 711 | 15 |
| 7 | Margarine, polyunsaturated, soft | 0.40 | 732 | 15 |
| 8 | Sugar, white, granulated | 0.47 | 406 | -100 |
| 9 | Rice, white, cooked | 0.47 | 121 | 22 |
| 10 | Samp and beans, 1:1, cooked | 0.47 | 124 | 41 |
| 11 | Sugar, brown | 0.51 | 399 | -97 |
| 12 | Rice, brown, cooked | 0.55 | 112 | 27 |
| 13 | Popcorn, plain | 0.65 | 521 | 16 |
| 14 | Salad dressing, mayonnaise | 0.70 | 517 | -11 |
| 15 | Pasta, Macaroni/Spaghetti, cooked | 0.77 | 157 | 31 |
| 16 | White bread (fortified) | 0.81 | 247 | 79 |
| 17 | Oats, rolled, cooked | 0.83 | 67 | 28 |
| 18 | Brown bread (fortified) | 0.83 | 246 | 108 |
| 19 | Sugar beans, cooked | 1.09 | 147 | 78 |
| 20 | Lentils, split, cooked | 1.09 | 122 | 73 |
| 21 | Potato, boiled without skin | 1.11 | 76 | 38 |
| 22 | Peanut butter, smooth style | 1.16 | 655 | 8 |
| 23 | Roti, made with sun oil | 1.21 | 594 | 6 |
| 24 | Lentils, whole, cooked | 1.23 | 121 | 104 |
| 25 | Polony / Bologna, beef and pork | 1.34 | 301 | 3 |
| 26 | Ice cream, regular (10% fat) | 1.34 | 207 | -13 |
| 27 | Macaroni/Spaghetti, wholewheat, cooked | 1.38 | 134 | 34 |
| 28 | Cookies, commercial, plain | 1.40 | 461 | -11 |
| 29 | Cookies, commercial, with filling | 1.40 | 487 | -18 |
| 30 | Corn flakes, plain, breakfast cereal | 1.43 | 383 | 71 |
| 31 | Snack, savoury, average, e.g. Niknaks, Fritos^ǂ^ | 1.46 | 556 | 1 |
| 32 | Peanut butter (unsalted/unsweetened) | 1.48 | 568 | 12 |
| 33 | Muffin, plain | 1.50 | 309 | 8 |
| 34 | Salad dressing, French | 1.51 | 578 | -5 |
| 35 | Sweets, hard boiled and soft jelly type | 1.54 | 383 | -48 |
| 36 | Butter | 1.63 | 726 | -20 |
| 37 | Weet-Bix, breakfast cereal | 1.70 | 371 | 33 |
| 38 | Vetkoek, home-made^≠^ | 1.73 | 246 | 25 |
| 39 | Jam/Marmalade | 1.81 | 286 | -40 |
| 40 | Cold drink, carbonated | 1.83 | 41 | -98 |
| 41 | All bran flakes, breakfast cereal | 1.92 | 374 | 105 |
| 42 | Potato chips/French fries, fried in sunflower oil | 1.92 | 312 | 12 |
| 43 | Morvite, original instant porridge^¶^ | 2.01 | 64 | 49 |
| 44 | Maas/Sour milk | 2.02 | 64 | 34 |
| 45 | Milk, full fat/whole, fresh | 2.08 | 63 | 34 |
| 46 | Pie, chicken, commercial, baked | 2.10 | 379 | 23 |
| 47 | Noodles, egg, cooked | 2.11 | 139 | 36 |
| 48 | Milk, full fat/whole, UHT | 2.16 | 63 | 30 |
| 49 | Chicken, feet, raw | 2.18 | 204 | 18 |
| 50 | Peanuts, roasted, salted | 2.19 | 640 | 17 |
| 51 | Doughnut, plain | 2.25 | 421 | -2 |
| 52 | Onion, boiled | 2.28 | 46 | 57 |
| 53 | Baked beans, canned in tomato sauce | 2.31 | 108 | 38 |
| 54 | Squash, butternut, boiled | 2.41 | 56 | 117 |
| 55 | Snack, savoury, potato crisps/chips | 2.41 | 553 | 29 |
| 56 | Vienna sausage, beef and pork, canned^§^ | 2.46 | 225 | -8 |
| 57 | Milk, low fat/2% fat, fresh | 2.55 | 51 | 41 |
| 58 | Avocado, raw (peeled) | 2.58 | 244 | 13 |
| 59 | Dairy-fruit juice mix | 2.65 | 62 | 18 |
| 60 | Sausage, beef & pork / boerewors, grilled | 2.65 | 387 | -10 |
| 61 | Samoosa, with mutton filling | 2.67 | 597 | -3 |
| 62 | Apple, golden delicious, raw | 2.85 | 66 | 25 |
| 63 | Pear, raw | 2.89 | 73 | 24 |
| 64 | Egg, chicken, whole, boiled/poached | 3.02 | 147 | 131 |
| 65 | Yoghurt, plain, double cream | 3.05 | 100 | 44 |
| 66 | Sweets, chocolate, milk | 3.05 | 550 | -25 |
| 67 | Carrot, boiled | 3.21 | 39 | 169 |
| 68 | Orange-fleshed sweet potato baked with skin | 3.25 | 94 | 150 |
| 69 | Chicken, giblets, cooked | 3.30 | 150 | 193 |
| 70 | Orange, raw (peeled) | 3.34 | 54 | 57 |
| 71 | Banana, raw (peeled) | 3.52 | 91 | 47 |
| 72 | Bacon, cured, pan-fried/grilled | 3.53 | 525 | 1 |
| 73 | Yoghurt, fruit, low fat, sweetened | 3.61 | 90 | 17 |
| 74 | Chicken, meat and skin, frozen, roasted | 3.62 | 219 | 35 |
| 75 | Beetroot, boiled with skin | 3.66 | 52 | 51 |
| 76 | Beef, brisket / regular mince, cooked | 3.91 | 341 | 17 |
| 77 | Soya mince, cooked | 3.95 | 99 | 126 |
| 78 | Patty, beef, frozen, grilled | 4.02 | 288 | 30 |
| 79 | White-fleshed sweet potato, boiled without skin | 4.15 | 74 | 27 |
| 80 | Cheese, Cheddar | 4.15 | 393 | 29 |
| 81 | Cheese, processed, full fat | 4.17 | 327 | 10 |
| 82 | Mixed vegetables, frozen, boiled | 4.22 | 64 | 135 |
| 83 | Cheese, Gouda (Edam, Swiss) | 4.51 | 377 | 31 |
| 84 | Sweets, fruit gum | 4.79 | 364 | -34 |
| 85 | Peas, frozen, boiled | 4.91 | 84 | 86 |
| 86 | Yoghurt, plain, low fat | 5.00 | 61 | 49 |
| 87 | Naartjie/Tangerine, raw (peeled) | 5.51 | 55 | 44 |
| 88 | Pork, loin, grilled (chop) | 5.55 | 234 | 48 |
| 89 | Fish, medium fat, grilled/steamed | 5.91 | 167 | 57 |
| 90 | Chicken, white meat, fresh, cooked | 6.02 | 152 | 49 |
| 91 | Pilchard in tomato sauce, canned | 6.10 | 127 | 162 |
| 92 | Beef, chuck, cooked | 6.65 | 242 | 56 |
| 93 | Plum, raw | 6.82 | 55 | 26 |
| 94 | Peach, raw | 6.99 | 47 | 29 |
| 95 | Mutton, shoulder, braised | 7.08 | 334 | 22 |
| 96 | Cabbage, boiled | 7.47 | 25 | 86 |
| 97 | Cold drink, squash, diluted | 7.58 | 28 | -45 |
| 98 | Beef, topside /lean mince, cooked | 7.87 | 218 | 60 |
| 99 | Nectarine, raw | 7.96 | 53 | 25 |
| 100 | Mutton, loin, grilled (chop) | 8.12 | 306 | 16 |
| 101 | Mango, raw (peeled) | 8.14 | 72 | 45 |
| 102 | Tomato, raw | 9.54 | 22 | 115 |
| 103 | Gem squash, boiled | 9.75 | 23 | 80 |
| 104 | Grape, average, raw | 9.90 | 72 | 24 |
| 105 | Green beans, frozen, boiled | 10.03 | 38 | 92 |
| 106 | Mango and orange juice | 10.59 | 56 | 34 |
| 107 | Fish, low fat, grilled | 11.33 | 105 | 167 |
| 108 | Pumpkin, boiled | 11.85 | 22 | 173 |
| 109 | Cauliflower, boiled | 15.47 | 22 | 92 |
| 110 | Spinach (Swiss Chard), boiled | 15.58 | 32 | 231 |
| 111 | Broccoli, boiled | 15.80 | 28 | 135 |
| 112 | Pepper, sweet, green, boiled | 20.14 | 38 | 132 |
| 113 | Tuna, canned in water | 20.97 | 87 | 103 |
| 114 | Lettuce, raw | 27.52 | 16 | 134 |
| 115 | Cucumber, English, raw | 40.16 | 15 | 89 |
| 116 | Pineapple, raw (peeled) | 48.98 | 59 | 32 |

ZAR, South African rands; NRF, nutrient rich food; 100kcal = 418kJ

^*^ Samp - dried corn kernels

^ǂ^ Niknaks, Fritos - Corn-based snack

^≠^ Vetkoek - Fried dough bread

^¶^ Morvite - instant sorghum porridge

^§^ Vienna sausage - Hot dog / Frankfurter (thin parboiled sausage traditionally made of pork and beef)

**Table** **S2**. Ranking selected South African foods according to nutrient-to-price ratio per price per 100kcal. Also shown are NRF9.3 scores and price per 100kcal.

| No. | Food | Nutrient density relative to cost (NRF9.3/price 100kcal) | | Nutrient density (NRF9.3/100kcal) | | Price per 100kcal | |
| --- | --- | --- | --- | --- | --- | --- | --- |
| 1 | Soft porridge (maize meal-fortified) | 242 | 44 | | 0.18 | |  |
| 2 | Stiff porridge (maize meal-fortified) | 242 | 44 | | 0.18 | |  |
| 3 | Brown bread (fortified) | 129 | 108 | | 0.83 | |  |
| 4 | Samp, cooked (white)^*^ | 102 | 26 | | 0.26 | |  |
| 5 | White bread (fortified) | 98 | 79 | | 0.81 | |  |
| 6 | Samp and beans, 1:1, cooked | 87 | 41 | | 0.47 | |  |
| 7 | Lentils, Whole, cooked | 85 | 104 | | 1.23 | |  |
| 8 | Sugar beans, cooked | 71 | 78 | | 1.09 | |  |
| 9 | Lentils, split, cooked | 67 | 73 | | 1.09 | |  |
| 10 | Chicken giblets, cooked (simmered) | 58 | 193 | | 3.30 | |  |
| 11 | All bran flakes, breakfast cereal | 55 | 105 | | 1.92 | |  |
| 12 | Carrot, boiled (flesh and skin) | 53 | 169 | | 3.21 | |  |
| 13 | Corn flakes, plain, breakfast cereal | 49 | 71 | | 1.43 | |  |
| 14 | Brown rice, cooked | 49 | 27 | | 0.55 | |  |
| 15 | Butternut, squash, boiled | 49 | 117 | | 2.41 | |  |
| 16 | White rice, cooked | 47 | 22 | | 0.47 | |  |
| 17 | Orange fleshed sweet potato, baked with skin | 46 | 150 | | 3.25 | |  |
| 18 | Egg, chicken, boiled/poached | 43 | 131 | | 3.02 | |  |
| 19 | Pasta, Macaroni/Spaghetti, cooked | 40 | 31 | | 0.77 | |  |
| 20 | Margarine, brick/hard | 40 | 15 | | 0.37 | |  |
| 21 | Margarine, polyunsaturated, soft | 38 | 15 | | 0.40 | |  |
| 22 | Potato, boiled without skin | 34 | 38 | | 1.11 | |  |
| 23 | Oats, rolled, cooked | 34 | 28 | | 0.83 | |  |
| 24 | Mixed vegetables, frozen, boiled | 32 | 135 | | 4.22 | |  |
| 25 | Soya mince, cooked | 32 | 126 | | 3.95 | |  |
| 26 | Pilchards in tomato sauce, canned | 27 | 162 | | 6.10 | |  |
| 27 | Popcorn, plain | 25 | 16 | | 0.65 | |  |
| 28 | Pasta, whole wheat Macaroni/Spaghetti, cooked | 25 | 34 | | 1.38 | |  |
| 29 | Onion, boiled | 25 | 57 | | 2.28 | |  |
| 30 | Morvite original instant porridge, prepared^¶^ | 25 | 49 | | 2.01 | |  |
| 31 | Weet-Bix, breakfast cereal | 19 | 33 | | 1.70 | |  |
| 32 | Peas, frozen, boiled | 18 | 86 | | 4.91 | |  |
| 33 | Orange, raw (peeled) | 17 | 57 | | 3.34 | |  |
| 34 | Noodles, egg, cooked | 17 | 36 | | 2.11 | |  |
| 35 | Maas/Sour milk | 17 | 34 | | 2.02 | |  |
| 36 | Baked beans, canned in tomato sauce | 17 | 38 | | 2.31 | |  |
| 37 | Milk, low fat/2% fat, fresh | 16 | 41 | | 2.55 | |  |
| 38 | Milk, full fat/whole, fresh | 16 | 34 | | 2.08 | |  |
| 39 | Spinach (Swiss Chard), boiled | 15 | 231 | | 15.58 | |  |
| 40 | Fish, low fat, grilled | 15 | 167 | | 11.33 | |  |
| 41 | Pumpkin, boiled | 15 | 173 | | 11.85 | |  |
| 42 | Yoghurt, plain, double cream | 15 | 44 | | 3.05 | |  |
| 43 | Vetkoek, home-made^≠^ | 14 | 25 | | 1.73 | |  |
| 44 | Beetroot, boiled with skin | 14 | 51 | | 3.66 | |  |
| 45 | Milk, full fat/whole, UHT | 14 | 30 | | 2.16 | |  |
| 46 | Banana, raw (peeled) | 13 | 47 | | 3.52 | |  |
| 47 | Snack, savoury, potato crisps/chips | 12 | 29 | | 2.41 | |  |
| 48 | Tomato, raw | 12 | 115 | | 9.54 | |  |
| 49 | Cabbage, boiled | 12 | 86 | | 7.47 | |  |
| 50 | Pie, chicken, commercial, baked | 11 | 23 | | 2.10 | |  |
| 51 | Chicken, meat and skin, frozen, roasted | 10 | 35 | | 3.62 | |  |
| 52 | Yoghurt, plain, low fat | 10 | 49 | | 5.00 | |  |
| 53 | Fish, medium fat, grilled/steamed | 10 | 57 | | 5.91 | |  |
| 54 | Green beans, frozen, boiled | 9 | 92 | | 10.03 | |  |
| 55 | Apple, Golden Delicious, raw | 9 | 25 | | 2.85 | |  |
| 56 | Pork, loin, grilled (chop) | 9 | 48 | | 5.55 | |  |
| 57 | Broccoli, boiled | 9 | 135 | | 15.80 | |  |
| 58 | Beef, chuck, cooked | 8 | 56 | | 6.65 | |  |
| 59 | Chicken, feet, raw | 8 | 18 | | 2.18 | |  |
| 60 | Gem squash, boiled | 8 | 80 | | 9.75 | |  |
| 61 | Pear, raw | 8 | 24 | | 2.89 | |  |
| 62 | Chicken, white meat, fresh, cooked | 8 | 49 | | 6.02 | |  |
| 63 | Peanut butter (unsalted/unsweetened) | 8 | 12 | | 1.48 | |  |
| 64 | Naartjie/Tangerine, raw (peeled) | 8 | 44 | | 5.51 | |  |
| 65 | Peanuts, roasted, salted | 8 | 17 | | 2.19 | |  |
| 66 | Beef, topside /lean mince, cooked | 8 | 60 | | 7.87 | |  |
| 67 | Patty, beef, frozen, grilled | 7 | 30 | | 4.02 | |  |
| 68 | Peanut butter, smooth style | 7 | 8 | | 1.16 | |  |
| 69 | Cheese, Cheddar | 7 | 29 | | 4.15 | |  |
| 70 | Cheese, Gouda (Edam, Swiss) | 7 | 31 | | 4.51 | |  |
| 71 | Dairy-fruit juice mix | 7 | 18 | | 2.65 | |  |
| 72 | Pepper, sweet, green, boiled | 7 | 132 | | 20.14 | |  |
| 73 | Potato chips/French fries | 6 | 12 | | 1.92 | |  |
| 74 | White-fleshed sweet potato, boiled | 6 | 27 | | 4.15 | |  |
| 75 | Cauliflower, boiled | 6 | 92 | | 15.47 | |  |
| 76 | Mango, raw (peeled) | 6 | 45 | | 8.14 | |  |
| 77 | Muffin, plain | 5 | 8 | | 1.50 | |  |
| 78 | Avocado, raw (peeled) | 5 | 13 | | 2.58 | |  |
| 79 | Tuna, canned in water | 5 | 103 | | 20.97 | |  |
| 80 | Lettuce, raw | 5 | 134 | | 27.52 | |  |
| 81 | Yoghurt, fruit, low fat, sweetened | 5 | 17 | | 3.61 | |  |
| 82 | Roti, made with sun oil | 5 | 6 | | 1.21 | |  |
| 83 | Beef, brisket / regular mince, cooked | 4 | 17 | | 3.91 | |  |
| 84 | Peach, raw | 4 | 29 | | 6.99 | |  |
| 85 | Plum, raw | 4 | 26 | | 6.82 | |  |
| 86 | Mango and orange juice | 3 | 34 | | 10.59 | |  |
| 87 | Nectarine, raw | 3 | 25 | | 7.96 | |  |
| 88 | Mutton, shoulder, braised | 3 | 22 | | 7.08 | |  |
| 89 | Cheese, processed, full fat | 3 | 10 | | 4.17 | |  |
| 90 | Grape, average, raw | 3 | 25 | | 9.90 | |  |
| 91 | Polony / Bologna, beef and pork | 2 | 3 | | 1.34 | |  |
| 92 | Cucumber, English, raw (flesh and skin) | 2 | 89 | | 40.16 | |  |
| 93 | Mutton, loin, grilled (chop) | 2 | 16 | | 8.12 | |  |
| 94 | Snack, savoury, average, e.g., Niknaks, Fritos^ǂ^ | 1 | 1 | | 1.46 | |  |
| 95 | Pineapple, raw (peeled) | 0.7 | 32 | | 48.98 | |  |
| 96 | Bacon, cured, pan-fried/grilled | 0.4 | 1 | | 3.53 | |  |
| 97 | Doughnut, plain | -1 | -2 | | 2.25 | |  |
| 98 | Samoosa, with mutton filling | -1 | -3 | | 2.67 | |  |
| 99 | Vienna sausage, beef and pork, canned^§^ | -3 | -8 | | 2.46 | |  |
| 100 | Salad dressing, French | -3 | -5 | | 1.51 | |  |
| 101 | Sausage, beef & pork / boerewors, grilled | -4 | -10 | | 2.65 | |  |
| 102 | Cold drink, squash, diluted | -6 | -45 | | 7.58 | |  |
| 103 | Sweets, fruit gum | -7 | -34 | | 4.79 | |  |
| 104 | Cookies, commercial, plain | -8 | -11 | | 1.40 | |  |
| 105 | Sweets, chocolate, milk | -8 | -25 | | 3.05 | |  |
| 106 | Ice cream, regular (10% fat) | -10 | -13 | | 1.34 | |  |
| 107 | Canola oil | -11 | -4 | | 0.35 | |  |
| 108 | Butter | -12 | -20 | | 1.63 | |  |
| 109 | Cookies, commercial, with filling | -13 | -18 | | 1.40 | |  |
| 110 | Salad dressing, mayonnaise | -16 | -11 | | 0.70 | |  |
| 111 | Sunflower oil | -18 | -5 | | 0.30 | |  |
| 112 | Jam/Marmalade | -22 | -40 | | 1.81 | |  |
| 113 | Sweets, hard boiled and soft jelly type | -31 | -48 | | 1.54 | |  |
| 114 | Cold drink, carbonated | -54 | -98 | | 1.83 | |  |
| 115 | Sugar, brown | -189 | -97 | | 0.51 | |  |
| 116 | Sugar, white, granulated | -213 | -100 | | 0.47 | |  |

NRF, nutrient rich food; 100kcal = 418kJ

^*^ Samp - dried corn kernels

^¶^ Morvite - instant sorghum porridge

^≠^ Vetkoek - Fried dough bread.

^ǂ^ Niknaks, Fritos - Corn-based snack

^§^ Vienna sausage - Hot dog / Frankfurter (thin parboiled sausage traditionally made of pork and beef)

**Table S3**. Ranking selected South African foods according to nutrient-to-price ratio per price per 100g. Also shown are NRF9.3 scores and price per 100g.

| No. | Food | Nutrient density relative to cost (NRF9.3/price 100g) | Nutrient density (NRF9.3/100g) | Price per 100g |
| --- | --- | --- | --- | --- |
| 1 | Soft porridge (maize meal-fortified) | 236 | 65 | 0.28 |
| 2 | Stiff porridge (maize meal-fortified) | 199 | 28 | 0.14 |
| 3 | Carrot, boiled (flesh and skin) | 102 | 127 | 1.24 |
| 4 | Samp, cooked (white)^*^ | 102 | 27 | 0.27 |
| 5 | Brown bread (fortified) | 98 | 202 | 2.05 |
| 6 | Samp and beans, 1:1, cooked | 87 | 51 | 0.59 |
| 7 | Lentils, Whole, cooked | 85 | 126 | 1.49 |
| 8 | White bread (fortified) | 82 | 164 | 2.00 |
| 9 | Sugar beans, cooked | 71 | 114 | 1.60 |
| 10 | Lentils, Split, cooked | 67 | 89 | 1.33 |
| 11 | All bran flakes, breakfast cereal | 50 | 355 | 7.16 |
| 12 | Brown rice, cooked | 49 | 30 | 0.61 |
| 13 | Butternut, squash, boiled | 49 | 66 | 1.35 |
| 14 | Chicken giblets, cooked | 48 | 239 | 4.96 |
| 15 | Orange fleshed sweet potato, baked with skin | 48 | 146 | 3.04 |
| 16 | White rice, cooked | 47 | 27 | 0.57 |
| 17 | Corn flakes, plain, breakfast cereal | 42 | 232 | 5.47 |
| 18 | Pasta, Macaroni/Spaghetti, cooked | 40 | 48 | 1.20 |
| 19 | Potato, boiled without skin | 34 | 29 | 0.84 |
| 20 | Oats, rolled, cooked | 34 | 19 | 0.55 |
| 21 | Egg, chicken, boiled/poached | 33 | 146 | 4.44 |
| 22 | Mixed vegetables, frozen, boiled | 32 | 86 | 2.69 |
| 23 | Soya mince, cooked | 32 | 126 | 3.93 |
| 24 | Margarine, brick/hard | 32 | 83 | 2.60 |
| 25 | Margarine, polyunsaturated, soft | 32 | 93 | 2.93 |
| 26 | Popcorn, plain | 25 | 85 | 3.37 |
| 27 | Pasta, whole wheat Macaroni/Spaghetti, cooked | 25 | 46 | 1.85 |
| 28 | Onion, boiled | 25 | 26 | 1.05 |
| 29 | Morvite original instant porridge, prepared^¶^ | 25 | 32 | 1.28 |
| 30 | Pilchards in tomato sauce, canned | 23 | 179 | 7.74 |
| 31 | Weet-Bix, breakfast cereal | 19 | 122 | 6.30 |
| 32 | Peas, frozen, boiled | 18 | 72 | 4.12 |
| 33 | Orange, raw (peeled) | 17 | 31 | 1.82 |
| 34 | Noodles, egg, cooked | 17 | 50 | 2.92 |
| 35 | Spinach (Swiss Chard), boiled | 17 | 84 | 4.97 |
| 36 | Maas/Sour milk | 17 | 22 | 1.30 |
| 37 | Baked beans, canned in tomato sauce | 17 | 41 | 2.50 |
| 38 | Pumpkin, boiled | 16 | 43 | 2.61 |
| 39 | Milk, low fat/2% fat, fresh | 16 | 21 | 1.30 |
| 40 | Milk, full fat/whole, fresh | 16 | 21 | 1.30 |
| 41 | Yoghurt, plain, double cream | 15 | 44 | 3.03 |
| 42 | Fish, low fat, grilled | 14 | 171 | 11.93 |
| 43 | Vetkoek, home-made^≠^ | 14 | 60 | 4.24 |
| 44 | Beetroot, boiled with skin | 14 | 27 | 1.89 |
| 45 | Milk, full fat/whole, UHT | 14 | 19 | 1.35 |
| 46 | Banana, raw (peeled) | 13 | 43 | 3.21 |
| 47 | Tomato, raw | 12 | 25 | 2.07 |
| 48 | Cabbage, boiled | 12 | 21 | 1.85 |
| 49 | Pie, chicken, commercial, baked | 11 | 87 | 7.95 |
| 50 | Chicken, meat and skin, frozen, roasted | 10 | 78 | 7.93 |
| 51 | Yoghurt, plain, low fat | 10 | 30 | 3.03 |
| 52 | Fish, medium fat, grilled/steamed | 10 | 94 | 9.86 |
| 53 | Green beans, frozen, boiled | 9 | 35 | 3.83 |
| 54 | Apple, Golden Delicious, raw | 9 | 17 | 1.88 |
| 55 | Pork, loin, grilled (chop) | 9 | 113 | 12.98 |
| 56 | Broccoli, boiled | 9 | 38 | 4.46 |
| 57 | Beef, chuck, cooked | 8 | 135 | 16.11 |
| 58 | Chicken, feet, raw | 8 | 36 | 4.44 |
| 59 | Gem squash, boiled | 8 | 18 | 2.26 |
| 60 | Pear, raw | 8 | 17 | 2.10 |
| 61 | Chicken, white meat, fresh, cooked | 8 | 75 | 9.18 |
| 62 | Peanut butter (unsalted/unsweetened) | 8 | 68 | 8.42 |
| 63 | Naartjie/Tangerine, raw (peeled) | 8 | 24 | 3.03 |
| 64 | Peanuts, roasted, salted | 8 | 110 | 14.00 |
| 65 | Beef, topside /lean mince, cooked | 8 | 132 | 17.16 |
| 66 | Patty, beef, frozen, grilled | 7 | 86 | 11.57 |
| 67 | Peanut butter, smooth style | 7 | 54 | 7.62 |
| 68 | Cheese, Cheddar | 7 | 115 | 16.31 |
| 69 | Cheese, Gouda (Edam, Swiss) | 7 | 117 | 17.00 |
| 70 | Dairy-fruit juice mix | 7 | 11 | 1.63 |
| 71 | Pepper, sweet, green, boiled | 7 | 50 | 7.55 |
| 72 | Potato chips/French fries | 6 | 39 | 5.99 |
| 73 | White-fleshed sweet potato, boiled without skin | 6 | 20 | 3.09 |
| 74 | Cauliflower, boiled | 6 | 21 | 3.47 |
| 75 | Mango, raw (peeled) | 6 | 33 | 5.90 |
| 76 | Muffin, plain | 5 | 25 | 4.64 |
| 77 | Avocado, raw (peeled) | 5 | 31 | 6.29 |
| 78 | Tuna, canned in water | 5 | 90 | 18.33 |
| 79 | Lettuce, raw | 5 | 21 | 4.38 |
| 80 | Yoghurt, fruit, low fat, sweetened | 5 | 16 | 3.23 |
| 81 | Roti, made with sun oil | 5 | 33 | 7.18 |
| 82 | Beef, brisket / regular mince, cooked | 4 | 59 | 13.33 |
| 83 | Peach, raw | 4 | 14 | 3.27 |
| 84 | Snack, savoury, potato crisps/chips | 4 | 54 | 13.33 |
| 85 | Plum, raw | 4 | 14 | 3.77 |
| 86 | Mango and orange juice | 3 | 19 | 5.90 |
| 87 | Nectarine, raw | 3 | 13 | 4.24 |
| 88 | Mutton, shoulder, braised | 3 | 73 | 23.61 |
| 89 | Cheese, processed, full fat | 3 | 34 | 13.61 |
| 90 | Grape, average, raw | 2 | 18 | 7.08 |
| 91 | Polony / Bologna, beef and pork | 2 | 9 | 4.03 |
| 92 | Cucumber, English, raw (flesh and skin) | 2 | 13 | 5.90 |
| 93 | Mutton, loin, grilled (chop) | 2 | 49 | 24.85 |
| 94 | Snack, savoury, average, e.g. Niknaks, Fritos^ǂ^ | 1 | 8 | 8.14 |
| 95 | Pineapple, raw (peeled) | 0.7 | 19 | 28.87 |
| 96 | Bacon, cured, pan-fried/grilled | 0.4 | 7 | 18.54 |
| 97 | Doughnut, plain | -1 | -9 | 9.47 |
| 98 | Samoosa, with mutton filling | -1 | -20 | 15.91 |
| 99 | Butter | -2 | -26 | 11.80 |
| 100 | Sweets, fruit gum | -3 | -53 | 17.46 |
| 101 | Vienna sausage, beef and pork, canned^§^ | -3 | -18 | 5.53 |
| 102 | Salad dressing, French (vinegar, sun oil) | -3 | -29 | 8.72 |
| 103 | Sausage, beef & pork / boerewors, grilled | -4 | -40 | 10.27 |
| 104 | Cold drink, squash, diluted | -6 | -13 | 2.15 |
| 105 | Cookies, commercial, plain | -7 | -50 | 6.44 |
| 106 | Sweets, chocolate, milk | -8 | -131 | 16.78 |
| 107 | Ice cream, regular (10% fat) | -10 | -27 | 2.78 |
| 108  109  110  111  112  113  114  115  116 | Canola oil  Cookies, commercial, with filling  Salad dressing, mayonnaise  Sweets, hard boiled and soft jelly type  Jam/Marmalade  Sunflower oil  Sugar, brown  Sugar, white, granulated  Cold drink, carbonated | -11  -13  -16  -17  -17  -18  -42  -52  -132 | -33  -86  -57  -98  -88  -47  -86  -100  -99 | 3.05  6.83  3.64  5.89  5.18  2.62  2.03  1.91  0.75 |

NRF, nutrient rich food

^*^ Samp - dried corn kernels

^¶^ Morvite - instant sorghum porridge

^≠^ Vetkoek - Fried dough bread.

^ǂ^ Niknaks, Fritos - Corn-based snack

^§^ Vienna sausage - Hot dog / Frankfurter (thin parboiled sausage traditionally made of pork and beef)
